# Answer: Heterotopic Heart Transplant History and Concepts Cannot Be Neglected - Witnessing the History and Learning with Previous Practices

**DOI:** 10.21470/1678-9741-2021-0956

**Published:** 2021

**Authors:** Fábio Antonio Gaiotto, Antonio Carlos de Almeida Barbosa-Filho, Davi Freitas Tenório, Samuel Padovani Steffen

**Affiliations:** 1 Cardiovascular Surgery Division, Instituto do Coração do Hospital das Clínicas da Faculdade de Medicina da Universidade de São Paulo (InCor-HCFMUSP), São Paulo, São Paulo, Brazil.; 2 Hospital Israelita Albert Einstein - Pavilhão Vick e Joseph Safra, São Paulo, São Paulo, Brazil.; 3 Centro Universitário CESMAC, Maceió, Alagoas, Brazil.

Dear Luciana and José Pedro, we appreciate your letter regarding our paper “Heterotopic Heart Transplantation as a Left Ventricular Biological Assistance: a New Two-Stage Method Proposal”^[1]^ and your interesting comments, however we disagree with some of the points made in several aspects, and we aim to clarify them.

First of all, we must emphasize that our method proposal is not, strictly speaking, a heterotopic heart transplantation, instead, the donor’s heart is set in heterotopic position aiming reduction of pulmonary vascular resistance so that, in an opportune time, when pulmonary pressure decreases, a second and final surgery is performed making a “twist”, as we will place the donor’s heart on orthotopic position. This idea is a development of the already stablished experience with mechanical assistance devices.

The paper entitled “Experience with Heterotopic Heart Transplantation in Patients with Elevated Pulmonary Vascular Resistance. Late Follow-up”^[2]^ is a great contribution to the previous literature and suggests the anastomosis of the donor’s pulmonary trunk to the recipient’s right pulmonary artery, instead of the recipient’s pulmonary trunk, as suggested by Barnard and Losman^[3]^, or to the right atrium, as Copeland^[4]^ published. It’s clear that this approach leaves the need of a conduit between both donor’s and recipient’s pulmonary trunks, but we reaffirm that this consists of both circulations in parallel, which has inherent risks as we wrote in our brief communication: “In Barnard’s technique for HHT, both circulations - right and left - are in parallel, which progressively turns the donor’s heart responsible for the entire blood flow and progressively reducing the activity of the recipient’s heart. This fact might lead to arrhythmias and other disastrous consequences, such as thrombus and embolus formation. Besides that, continuous dilation of the myocardium provides a favorable site for endocarditis. All these factors led to the discontinuation of Barnard’s technique.”.

Another point that should be clarified, we agree that the left ventricle is the main site of thrombus and we never stated the opposite, but we know that when both circulations are in parallel, the donated heart is responsible for both pulmonary and systemic circulations, which could lead to clot formation on the native heart as we described earlier, but not only, these could also lead to arrhythmias and infection, for example. Our intention when anastomosing the native superior vena cava to donor’s superior vena cava by an end-to-end fashion is to preserve the donor’s right ventricular function, considering that the right ventricle is a flow-dependent chamber and that the Copeland’s technique with an end-to-side anastomosis provided atrophy of this chamber, which was undesirable for our technique as in near future, after the first surgery, it will be responsible for pulmonary circulation when finished the second stage on orthotopic position, as we wrote: “Considering that the RV is a flow-dependent chamber, its function would be preserved, as it will receive all the flow from the total superior vena cava (SVC) venous return, contrasting the RV atrophy associated with the reduced flow from the side anastomosis of the original Copeland’s technique, and this benefit would be attained by the direct end-to-end anastomosis of the donor SVC to the recipient SVC close to the brachiocephalic veins (Figure 2A), while closing the donor inferior vena cava (IVC) and the recipient SVC near to the right atrium taking care to avoid lesion of the sinoatrial node.”.

We read your comments about the need of sufficient decrease of pulmonary vascular resistance to perform an orthotopic transplantation and that is the core of our research, such as your own paper^[2]^ suggests significant reduction of pulmonary pressure, and furthermore, your concern about coronary artery disease on the donor’s hearts has been taken in consideration since our initial idea, as some papers calls them “marginal” donor hearts, and it’s clear that those hearts promotes poor outcomes when used for heterotopic heart transplantation and the opposite occurs when using healthy hearts who does not fit in marginal criteria, as we wrote: “ The “marginal” donor hearts, which means hearts that suffered a long ischemia time and are at high risk of complications, for example: hearts who needed high inotropic support, previous cardiac arrest or arrhythmia, abnormal wall motion visualized on echocardiogram and/or an electrocardiogram suggestive of ischemia. It is worth mentioning that most donor hearts that present with those clinical scenarios are excluded for OHT donation. So, it is obvious to say that we will not use “marginal” hearts.”

Finally, your concern about clinical indication and clinical practice of our proposal method, we briefly explain that it is the point of our research, and two hearts are two organs to control, which one is healthy and the other, even with regression of pulmonary pressure, is still an organ that suffered with pulmonary hypertension and its disfunction could be catastrophic. We hope to achieve satisfactory results so this method will create a window of opportunity to a select group of patients with contraindication for an orthotopic heart transplant around the world. Both history or concepts were not neglected or forgotten, but considering the brief communication format limits, all the significant contributions to this challenging pathology could not be brought forward.

Thank you again for the letter and the opportunity to clarify such interesting concepts.

Sincerely,
